# *Drosophila* gene *tao-1* encodes proteins with and without a Ste20 kinase domain that affect cytoskeletal architecture and cell migration differently

**DOI:** 10.1098/rsob.140161

**Published:** 2015-01-14

**Authors:** Ralf Pflanz, Aaron Voigt, Toma Yakulov, Herbert Jäckle

**Affiliations:** 1 Abteilung Molekulare Entwicklungsbiologie, Max Planck Institut für Biophysikalische Chemie, Göttingen, Germany; 2 Department of Neurology, University Medical Centre Aachen, Aachen, Germany; 3 Renal Division, University Hospital Freiburg, Freiburg, Germany

**Keywords:** cytoskeletal architecture, germline cell migration, ectopic pole cell induction, Ste20 kinase

## Abstract

Tao-1, the single representative of the Sterile 20 kinase subfamily in *Drosophila*, is best known for destabilizing microtubules at the actin-rich cortex, regulating the cytoskeletal architecture of cells. More recently, Tao-1 was shown to act in the Salvador–Warts–Hippo pathway by phosphorylating Hippo, regulating cell growth as well as cell polarity. Here, we show that *tao-1* encodes two proteins, one with the Sterile 20 kinase domain (Tao-L) and one without it (Tao-S), and that they act in an antagonistic manner. Tao-L expression causes lamellipodia-like cell protrusions, whereas Tao-S expression results in filopodia-like structures that make cells stick to the surface they attach to. Ectopic Tao-1 expression in the anterior region of *Drosophila* embryos results in pole cell formation as normally observed at the posterior end. Tao-S expression causes primordial germ cells (PGCs) to adhere to the inner wall of the gut primordia and prevents proper transepithelial migration to the gonads. Conversely, RNAi knockdowns of Tao-1 cause disordered migration of PGCs out of the gut epithelium, their dispersal within the embryo and cell death. The results reveal a novel function of Tao-1 in cell migration, which is based on antagonistic activities of two proteins encoded by a single gene.

## 2. Introduction

Embryonic development is based on the position-specific differentiation of cells, regulated cell divisions and the migration of cells from one location to another. Cell migration is central to homoeostatic processes such as local immune responses and the repair of injured tissues, and it is based on reorganizations of the cellular cytoskeleton. This process is coordinated and controlled by extensive transient signals, yet the majority of the signals and the transduction pathways are still unknown. This is especially important considering that the failure of cells to migrate, or the migration of wrong cell types to the wrong place, causes developmental defects and disease.

In most metazoans, primordial germ cells (PGCs) migrate as individual cells through and along a variety of tissues to reach and join the mesoderm-derived somatic gonadal precursors [1,2]. Several components required for the formation of PGCs and their directed migration to the gonads have been identified (reviewed in [3–5]). However, information concerning the nature of the signals that initiate and mediate PGC migration, as well as on factors required to orchestrate the reorganization of the cytoskeleton in the migrating PGCs, is yet not fully established.

Migrating PGCs are characterized by an actin-rich cortex and lamellipodia-like cytoplasmic protrusions [6], and, as they migrate through the midgut epithelium, by pseudopodial cytoplasmic expansions [7]. Here, we show that the activity of Tao-1, initially identified as a conserved microtubule-associated serine–threonine protein kinase of the Sterile 20 (Ste20) subfamily [8–12], can cause such a switch in cytoskeletal architecture. Ste20 kinases have the ability to reduce microtubule stability, effectively controlling the dynamics of the functional interactions between the plus ends of microtubules and the actin-rich cell cortex [12]. Tao-1 was also shown to control tissue growth by regulating the Salvador–Warts–Hippo (SWH) pathway [13,14]. Tao-1 maintains chromosomal stability by facilitating proper congression of the chromosomes, demonstrating that *tao-1*-dependent microtubule regulatory pathways are important for resolving erroneous kinetochore–microtubule attachments [15]. In addition, Tao-1 has been shown to participate in apoptosis of pole cells by inducing the apoptosis regulator Sickle in the absence of Nanos, which suppresses apoptosis to permit proper germline development [16]. Although much is known by now about the various aspects of Tao-1 function in multiple biological processes, the mechanism of action and how Tao-1 ties into the different regulatory pathways are still not understood. In fact, there is no evidence to suggest that tissue growth by Tao-1-dependent regulation of the SWH pathway, microtubule stability and apoptosis are in any way linked, but it has been speculated that Tao-1 could act at the convergence point between mechanical tension that regulates microtubule polymerization, control of tissue growth and the SWH pathway [13,14].

Another untended aspect of *tao-1* activity is that the gene encodes two proteins: in addition to the protein that contains a Ste20 kinase domain (‘Tao-L’), the single *tao-1* gene of *Drosophila* also encodes a second, smaller protein which lacks the Ste20 kinase domain (‘Tao-S’). Both proteins derive from the two major transcripts of the gene, which are generated by differential transcription [16,17]. Here, we focus on the previously neglected function of Tao-S by tissue culture approaches as well as gain-of-function and loss-of-function experiments with developing embryos. The results show that expression of Tao-S and Tao-L cause filopodia-like cytoplasmic protrusions and microtubule-dependent cytoplasmic expansions, respectively. Tao-S acts as an antagonist of Tao-L both in tissue culture cells and in transgenic animals, indicating that the *tao-1* gene encodes two proteins with opposing functions on the cytoskeletal architecture. In early development, overexpression of Tao-S in the posterior pole region prevents the proper migration of the PGCs. Ectopic expression in the anterior region of the preblastoderm embryo causes the formation of additional, anteriorly positioned pole cells. Thus, the two proteins not only participate in an antagonistic manner in setting up the cytoplasmic architecture, but also share a second function, which is independent of the Ste20 kinase domain. We also report a genetic interaction of Tao-1 and the G protein-coupled receptor (GPCR) Tre1, previously shown to be essential for initiating transepithelial migration of the PGCs [18].

## 3. Results

### 3.1. Expression of Tao-1 during embryogenesis and subcellular localization

**Figure 1.**
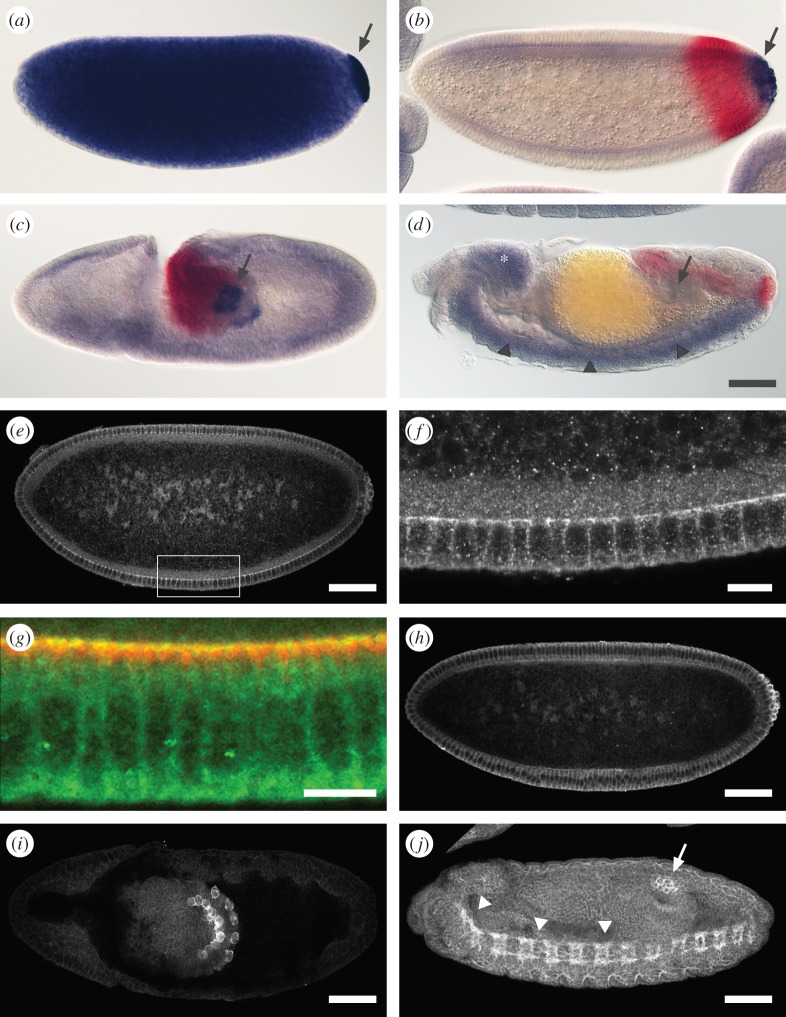
*tao-1* mRNA and protein distribution in early *Drosophila* development. (*a*–*d*) Accumulation of *tao-1* transcripts during early *Drosophila* development as visualized by RNA *in situ* hybridization using probes which detect Tao-L and Tao-S transcripts (blue staining). *brachyenteron* (*byn*) expression (red staining) serves as a molecular landmark for hindgut development. (*a*) Preblastoderm and (*b*) blastoderm embryos showing ubiquitous *tao-1* mRNA and its enrichment in pole plasm (arrow in *a*) and in pole cells (arrow in *b*). Note that Tao-S transcripts are degraded immediately after the pole cells have formed [16]. (*c*,*d*) During gastrulation, *tao-L* mRNA remains in PGCs at the onset of transepithelial migration (arrow in *c*) and when they are embedded in gonadal mesoderm (arrow in *d*), and it accumulates in the developing brain (asterisk in *d*) and the central nervous system (arrowheads in *d*). (*e*–*j*) Tao-L staining using mouse polyclonal antibodies. (*e*) During cellularization, Tao-L is present in low levels throughout the embryo and in pole cells. (*f*,*g*) Enlargements showing that Tao-1 is enriched at the tips of the infolding membranes. Merged image (*f*) shows double staining of Tao-L (green) and the basal membrane marker Disc Lost (Dlt) (red [19]). (*h*) After blastoderm cellularization, Tao-L is highly enriched in pole cells. (*i*) Dorsal view showing that, during gastrulation and the beginning transepithelial migration of PGCs, Tao-1 is exclusively found in PGCs. (*j*) At mid-stages of embryogenesis, Tao-1 appears at low levels in all cells of the embryo, remains highly enriched in PGCs that reached the gonadal mesoderm (arrow) and is highly expressed in the developing central nervous (arrowheads in *j*). Scale bars: (*e*,*f*,*h*–*j*) 50 µm; (*g*) 10 µm. Anterior is to the left, dorsal is upwards, except in (*i*) dorsal view and (*j*) oblique ventral view.

The *tao-1* gene of *Drosophila*, which encodes the single member of the Ste20 serine–threonine kinase protein family, is located close to the centromere in region 18D of the *Drosophila* X chromosome. As reported earlier, it encodes two different transcripts (electronic supplementary material, figure S1) under the control of two separate promoter regions [16]. The longer 4.8 kb transcript codes for a 1039 amino acid protein (‘Tao-L’) that contains the Ste20 kinase domain in the N-terminal region. The shorter 2.5 kb transcript encodes a 492 amino acid protein (‘Tao-S’) that lacks this domain. Figure 1 summarizes the expression patterns of *tao-1* and the localization of Tao-1 protein during embryonic development. *tao-1* transcripts are maternally expressed, ubiquitously distributed in the egg and early embryo (figure 1*a*), and enriched in the germ plasm at the posterior pole region of the early embryo including the pole cells (figure 1*a*,*b*; see also [16]). The transcripts remain in the PGCs during their integration into the developing midgut pocket (figure 1*c*). When PGCs migrate through the midgut epithelium to target the gonad precursors they continue to express Tao-1 (figure 1*d*; see also [16]). At the same time, transcripts accumulate also in the developing nervous system as a second site of embryonic *tao-1* expression (figure 1*d*). Note that *tao-S* trancripts are degraded immediately after pole cell formation. Thus, only *tao-L* transcripts are zygotically expressed and persist in the developing germ cells [16].

Using antibodies directed against the kinase domain of Tao-L, we found that Tao-L strongly accumulates at the leading edges of the inward-growing membranes that engulf the nuclei during blastoderm formation (figure 1*e*; enlarged in figure 1*f*,*g*) and most prominently in the pole cells (figure 1*h*). The transcripts and the protein stay in PGCs during gastrulation and transepithelial migration (figure 1*i*) until the PGCs reach the gonads (figure 1*j*). At this stage, Tao-1 was also detected in the central nervous system during mid-stages of embryogenesis (figure 1*j*). Taken together, the results show that during embryogenesis, Tao-1 is expressed in cells that migrate or grow over distances, such as PGCs, glia cells or axons.

**Figure 2.**
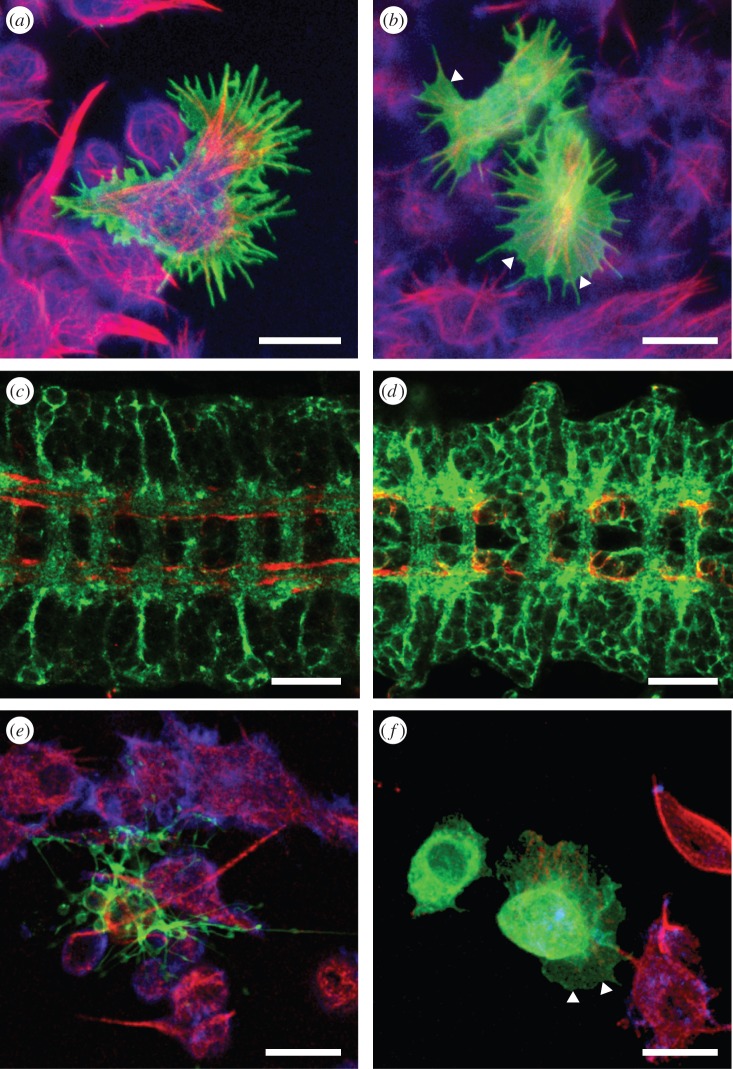
Cellular distribution of Tao-L and Tao-S, and their effects on cell shape and cell behaviour. UAS-dependent (*a*,*e*) Tao-S-GFP or (*b*,*f*) Tao-L-GFP were expressed in cultured *Drosophila* S2 cells in response to the cotransfected *actin5C*-GAL4 driver and in neuronal cells of the embryo in response to the *elav*-GAL4 driver (*c*,*d*; ventral view of the enlarged central nervous system, anterior is to the left). The microtubule networks in the cultured cells were visualized with anti-tubulin (red), filamentous actin with phalloidin (blue) and the longitudinal axonal tracks of the nervous system with anti-FasII antibodies (red). (*a*) Tao-S expression (green) in S2 cells 18 h after transfection. Note that Tao-S accumulates at the periphery of cells and causes formation of thin cell protrusions. (*b*) Tao-L expression (green) in S2 cells 18 h after transfection. The protein is ubiquitously expressed and causes the formation of lamellipodia-like protrusions (example indicated by white arrow heads). (*c*) Neural expression of Tao-S accumulates in axons. (*d*) Neural expression of Tao-L accumulates both in axons and the cell body cytoplasm of the neurons. (*e*) Tao-S expression (green) in S2 cells 24 h after transfection. Tao-S caused long and thin attached protrusions where the protein accumulates. Note that the cell body is below the focal plane and that the cells are firmly attached to the surface. (*f*) Tao-L expression (green) in S2 cells 24 h after transfection. Tao-L caused lamellipodia-like structures (white arrow heads). The microtubule network of the cells is strongly disrupted (red microtubule staining seen in the neighbouring, non-transfected control cells). Scale bars: (*a*,*b*,*e*,*f*) 10 µm; (*c*,*d*) 20 µm.

Next, we asked how the two Tao-1 proteins are distributed within the cells. As several attempts to generate antibodies specifically directed against Tao-S were unsuccessful, we examined the localization of GFP-tagged Tao-L or Tao-S in transfected Schneider S2 cells (figure 2). GFP-tagged Tao-S was predominantly found at the cell periphery (figure 2*a*), whereas GFP-tagged Tao-L was distributed throughout the cell cytoplasm and notably enriched at the leading edge of the lamellipodia-like structures (figure 2*b*). We also examined the cellular localization patterns in the embryo using GFP-tagged Tao-S and Tao-L transgene expression in response to the panneural *elav^C155^*-GAL4 driver in neurons [20]. We observed comparable cellular distribution patterns for the two proteins as observed in transfected Schneider S2 cells; that is, Tao-S was enriched in the periphery of the cells including the axons (figure 2*c*), whereas Tao-L is found in the cell bodies of the nervous system as well (figure 2*d*).

### 3.2. Expression of Tao-L and Tao-S result in different cell shape changes

Tao-L and Tao-S differ with respect to their N-terminal region where the Ste20 kinase domain is located. To test whether Tao-1 with and without potential kinase activity evoke the same or different cellular phenotypes, we transfected cultured Schneider S2 cells with transgenes expressing Tao-L and/or Tao-S protein, and examined the cells 18 and 24 h after transfection.

Eighteen hours after transfection, Tao-S expression had no discernible effect on microtubules, but the transfected cells developed long and thin filopodia-like structures (figure 2*a*) instead of the numerous lamellipodia-like structures observed after Tao-L expression (figure 2*b*; see also [12]). Six hours later (i.e. 24 h after transfection) the Tao-S-expressing cells had continued to extend their filopodia-like structures, resulting in ramifying structures (figure 2*e*). By contrast, Tao-L-expressing cells had developed very broad and prominent lamellipodia, and their microtubular network had dissolved (figure 2*f*). This Tao-L-dependent effect confirms that Tao-1 acts as a negative regulator of microtubule growth through the destabilization of microtubule plus ends [12]. In addition to the different cell shapes, the behaviour of Tao-S- and Tao-L-expressing cells were very different. Time-lapse confocal microscopy movies show that Tao-S expressing cells stall their movements and firmly attach to the substrate (electronic supplementary material, movie M1), whereas Tao-L expression caused large and highly dynamic cell protrusions, which constantly probe the environment (electronic supplementary material, movie M2).

To examine whether the different cellular phenotypes are due to the lack of kinase activity, we inactivated potential kinase function of Tao-L by replacing lysine 56 of the kinase domain by arginine. Expression of the mutated Tao-L protein (K56R) caused a Tao-S-like cellular phenotype (electronic supplementary material, figure S2). This result indicates the different phenotypes in response to Tao-L and Tao-S expression in cells are dependent on the presence and absence of the catalytic kinase domain in the two proteins.

**Figure 3.**
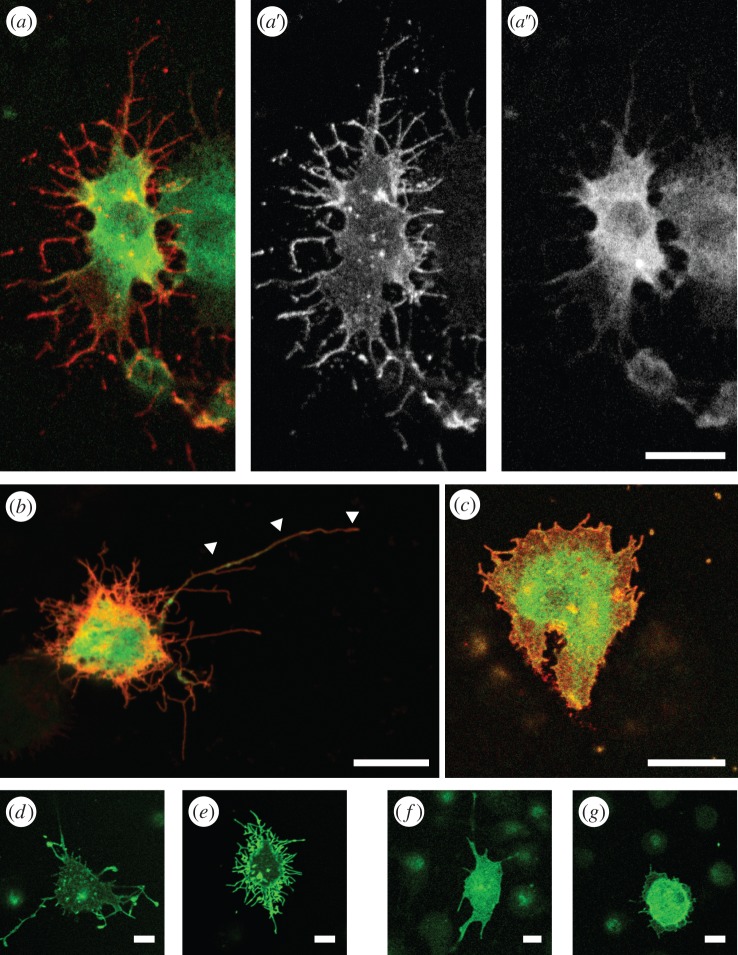
Tao-S and Tao-L activities are mediated by different cytoskeletal components. (*a*) UAS-dependent Tao-S-CFP (*a* red; separate channel in *a*′) and Tao-L-VENUS (*a* green; separate channel in *a*″) in response to the co-transfected *Actin5C*-GAL4 driver in *Drosophila* S2 cells. Tao-S accumulates in the cell cortex and distinctly in the cell protrusions (*a*′), whereas Tao-L is ubiquitously distributed throughout the cell cytoplasm (*a*″) as observed after the individual expression of the two proteins (see figure 2*a*,*b*,*e*,*f*). Co-expression of Tao-S and Tao-L results in an intermediate cellular phenotype compared to the Tao-S and Tao-L only expression (compare to figure 2*e*,*f*). (*b*) Expression of Tao-S-RFP (red) in the presence of GFP expression (control for the presence of a second UAS transgene, green) had no effect on the Tao-S-induced phenotype, i.e. cells produced the Tao-S-typical long and thin filopodia-like cell protrusions (arrowheads). Co-expression of Tao-L-GFP (*c*, green) results in reduced Tao-S-rich protrusions (red in *c*) formed around the Tao-L-type lamellipodia, and closer contact of cells to their substrate. (*d*) Cytochalasin D reduced the number of filopodia-like protrusions in Tao-S-expressing cells. (*e*) Vinblastine had no such effect on Tao-S expressing cells. (*f*) Cytochalasin D had no effect on Tao-L-dependent lamellipodia-like structures. (*g*) Vinblastine reduced the Tao-L-dependent lamellipodia-like structures. Scale bars in (*a*–*c*) represent 10 µm; in (*d*–*g*) 5 µm.

To test whether Tao-L and Tao-S act in an antagonistic manner, as suggested by the opposite phenotypes when expressed in cultured cells, we coexpressed VENUS-tagged Tao-L and ECFP-tagged Tao-S from transgenes driven by the *actin5C*-GAL4 transgene in Schneider S2 cells. Figure 3*a* shows that Tao-S is predominantly localized at the cellular edges, whereas Tao-L is found in the cytoplasm of the cell (see figure 3*a*′,*a*″; see also figure 2*a*,*b*). The cotransfected cells developed an intermediate cell shape phenotype (i.e. they produced both lamellipodia- and filopodia-like structures; figure 3*a*). The filopodia-like structures in response to Tao-S expression (figure 3*b*) were reduced in response to Tao-L expression (figure 3*c*). These results show that the two proteins encoded by Tao-1 have interdependent but different effects on cell shape and cell behaviour.

To see whether Tao-S interferes with Tao-L activity or whether it acts on different cytoskeleton components, we expressed either Tao-S or Tao-L in the Schneider S2 cells in the presence of drugs that specifically target either filamentous actin (F-actin) or microtubules (figure 3*d*–*g*). Cytochalasin D and latrunculin A, which interfere with F-actin stability and actin polymerization, inhibited the Tao-S-inducible filopodia-like structures, but did not interfere with the formation of Tao-L-dependent lamellipodia-like structures (figure 3*d*,*e*; electronic supplementary material, figure S3). Conversely, cells exposed to drugs that destabilize microtubules, such as nocodazole and vinblastine, inhibited lamellipodia-like structures in response to Tao-L expression, but had no effect on the formation of filopodia-like structures in response to Tao-S expression (figure 3*f*,*g*; electronic supplementary material, figure S3). These results indicate that Tao-L activity affects microtubule-mediated processes as shown earlier [12], whereas Tao-S affects actin-mediated processes.

### 3.3. *tao-1* has an essential function during fly development

In order to assess possible organismal effects caused by the lack of *tao-1* activity, we generated loss-of-function and temperature-sensitive mutant *tao-1* alleles, and performed RNAi knockdown experiments. Mutants were generated on the basis of four P-element insertions. Of the four P-element lines used to generate the mutants (electronic supplementary material, figure S1), EP(1)1455, GE(1)01525 and GE(1)02166 were homozygous viable, and GE(1)08166 was lethal. The vast majority of GE(1)08166 mutants died as pupae, but few hemizygous males survived to adulthood. Those individuals showed a strong paralytic phenotype before they died within a few days after hatching. Mobilization of the GE(1)08166-associated P-element resulted in revertants that were fully viable and fertile. This indicates that the P-element, which has been inserted close to the splice acceptor site of the second *tao-1* exon (electronic supplementary material, figure S1), was the cause of lethality.

To obtain genomic deletions of the *tao-1* locus, we performed imprecise P-element excision experiments with each of the four original P-element lines. We obtained an amorphic mutation (*tao50*) that has the first exon of the Tao-L transcript deleted (electronic supplementary material, figure S1) [17] and the temperature-sensitive hypomorphic allele *tao16* (electronic supplementary material, figure S1). Both mutants were rescued with a transgene that contained 19 kb of genomic DNA, which covers the coding region, 6 kb upstream and 4.5 kb downstream sequences of the *tao-1* gene (electronic supplementary material, figure S1). This result indicates that the P-element excision mutants only affect the *tao-1* gene function.

As *tao-1* is located in a position close to the centromere, we were unable to generate recombination events necessary for the generation of *tao-1* mutant germline clones. Therefore, we did not analyse *tao-1* mutants which lack the combined maternal and zygotic *tao-1* activities. However, we asked whether and when the lack of zygotic *tao-1* expression causes a mutant phenotype. Embryos lacking zygotic *tao-*1 activity developed into larvae that died. Lethality was also observed in response to zygotic *tao-1* RNAi expression from a transgene under the control of the ubiquitous *actin5C*-driver (see Material and methods) [20]. These observations establish that *tao-1* carries an essential zygotic function that cannot be compensated for by the maternal gene products (figure 1) provided by heterozygous females.

**Figure 4.**
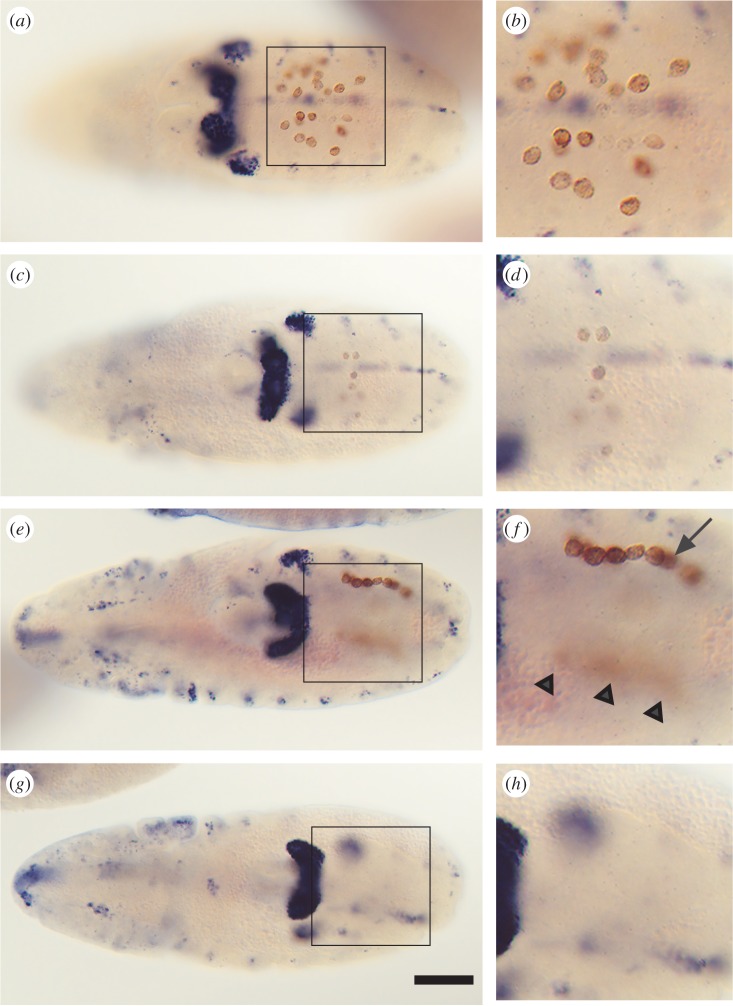
*tao-1* RNAi expression causes a reduction and dispersal of PGCs. Dorsal views of postgastrulation embryos showing Vasa expressing PGCs (brown) and, as a landmark, the developing malpighian tubules, brain and sensory neurons of the peripheral and central nervous system, which are stained with anti-Cut antibodies (blue). (*a*) Stage 10 wild-type embryo showing normal midgut transmigration of PGCs. (*b*) Enlargement of the boxed area in (*a*). (*c*) *tao-1* RNAi knockdown embryo at the same stage showing a strongly reduced number of migrating PGCs. Note that all Vasa expressing cells are in the focal plane. (*d*) Enlargement of the boxed area in (*c*). (*e*) Stage 12 wild-type embryo showing the normal attachment of PGCs to gonadal mesoderm precursors (focus on the PGC cluster on the right side of the embryo). (*f*) Enlargement of the boxed area in (*e*); arrow indicates the string of migrating PGCs, arrowheads point to the corresponding cluster of PGCs (out of focus) on the other side of the embryo. (g) Stage 12 *tao-1* RNAi knockdown embryo showing the lack of PGC clusters. (*h*) Enlargement of the boxed area in (*g*), where the PGC cluster should be observed if present. Anterior is to the left; scale bar, 50 µm.

Maternal *tao-1* transcripts are distributed throughout the egg and early embryo, and accumulate gradually and strongly in the posterior tip region (see figure 1*a*–*d*; see also [16]). In order to examine the need for of *tao-1* activity during early embryonic development, we expressed *tao-1* RNAi from an UAS-dependent transgene under control of the *actin5C* driver. Few embryos (about 5%) that received *tao-1* RNAi survived and developed into viable adult flies. However, the fertility of these escaper females was reduced by about 80%, as had been observed with mutant females that are homozygous for the weak *tao16* allele. In such females, the number of ovarioles was reduced. To test whether *tao16* mutant females contained fewer germline cells, and if so, when their number was reduced, we followed the fate of PGCs during development. Figure 4 shows that the number of PGCs that were visualized with antibodies directed against the germline-specific marker protein Vasa [21] was reduced in response to *tao-1* RNAi expression when control and RNAi-treated embryos were compared. In wild-type embryos at stage 10, PGCs migrate out of the midgut primordia towards the gonads (figure 4*a*,*b*). In *tao-1* RNAi-treated embryos of the same stage, the number of migrating PGCs is strongly reduced (to about 30%; figure 4*c*,*d*). At stage 12, when the wild-type PGCs form a string of migrating cells (figure 4*e*,*f*), no corresponding arrangement of PGCs was observed in the *tao-1* RNAi-treated embryos (figure 4*g*,*h*). This observation indicates that the loss of maternal *tao-1* activity affects either the production of pole cells or causes an early loss when PGCs migrate during early gastrulation.

### 3.4. Anterior Tao-1 expression causes ectopic pole cells

Pole cell formation at the posterior region of the early embryo depends on intensive local rearrangements of the cortical cytoskeletal architecture involving both actin- and microtubule-mediated events [22,23], reminiscent of the phenotypes observed after Tao-S and Tao-L expression in cultured cells. As both *tao-S* and *tao-L* transcripts are maternally expressed and highly enriched in the posterior region of the embryo when the pole cells are formed [16] (R.P. 2003, unpublished data), we asked whether increased levels of one or both of the *tao-1* transcripts may interfere with pole cell formation. We expressed GFP-fusions of Tao-L and Tao-S from UAS-containing transgenes in response to the maternal V3-GAL4 driver [24], and included the 3′ UTR sequences of the *nanos* transcript to localize the mRNAs in the posterior pole region of the early embryo [25]. Posterior expression of maternal Tao-L or Tao-S had no effect on the formation and the number of pole cells. However, elevated levels of *tao-S*, but not *tao-L*, had a strong effect on the migration of the PGCs.

**Figure 5.**
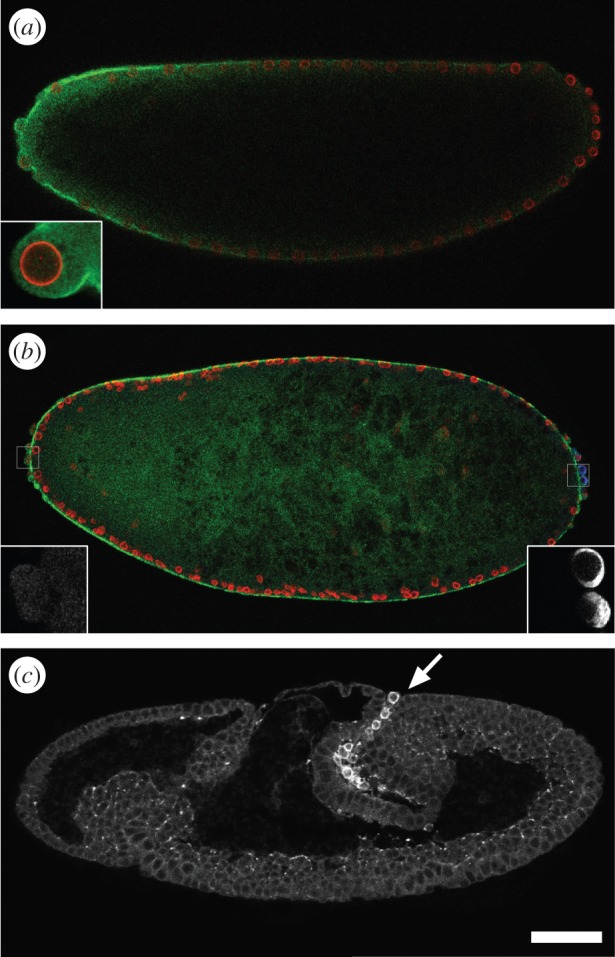
Anterior Tao-L expression induces ectopic pole cell formation. (*a*) Anterior expression of maternal *tao-L mRNA* (green) causes anterior pole cells. Anteriorly localized *tao-L* cDNA expression was provided from a transgene that contains the anterior localization 3′ UTR of *bicoid* (details in Material and methods). Preblastoderm nuclei are visualized with LaminO antibodies (red). Enlargement shows the budding-out of an anterior pole cell at the time when the normal pole cells form in the posterior region. (*b*) Tao-L (green) induced anterior pole cells lack the germline marker Vasa (left inset) that is present in posterior pole cells (right inset). (*c*) Gastrulating embryos that received maternal Tao-S (white) in the posterior region of the embryo develop a *tre1*-like mutant phenotype. Posterior localized Tao-S expression was provided from a transgene that contains the posterior localization 3′ UTR of *nanos* (details in Material and methods). Note that the PGCs are trapped in the combined midgut and hindgut primordium, and accumulate up to the surface of the embryo (arrow), and that they fail to cluster at the very tip region of the invaginating midgut primordium and to undergo transepithelial migration. For details see text. Scale bars: (*a*,*d*,*g*) 50 µm; (*c*,*f*) 10 µm.

We next asked whether maternal Tao-1 transcripts affect embryonic development when maternally expressed and positioned in an ectopic location of the syncytial preblastoderm embryo. To position *tao-L* and *tao-S* mRNA in the anterior pole region of the embryo, we added the 3′ UTR sequences of the gene *bicoid* [26]. Expression of Tao-L (figure 5*a*) and Tao-S (electronic supplementary material, figure S1) in the anterior region of the embryo resulted in the formation of ectopic ‘pole cells’ at the time when pole cells are normally formed at the posterior end of the embryo. Both the budding-out and the pinching-off processes occurred in parallel and indistinguishably from the normal pole cell formation at the posterior pole of the embryo. However, the anteriorly induced ectopic pole cells lack the molecular signature of PGCs such as the expression of the marker protein Vasa (figure 5*b*). Furthermore, the anterior pole cells remained in the position where they were generated. These results indicate that Tao-L and Tao-S are both capable of inducing pole cell formation in the early embryo, although they act in microtubule- and actin-mediated events, respectively.

### 3.5. Tao-S affects primordial germ cell migration

Enhanced maternal Tao-S expression in the posterior region of the embryo did not affect the formation and number of pole cells. However, when embryos entered gastrulation, the migration behaviour of the pole cells was strongly disturbed. The pole cells that obtained an extra load of maternal Tao-S failed to cluster and to enter the midgut pocket in a coordinated manner during the amnioproctodeal invagination (figure 5*c*). A variable number of PGCs remained outside the embryo (5–10%), and most of the PGCs did not reach the tip of the midgut primordium (more than 70%). Furthermore, PGCs failed to migrate through the epithelium in a coordinated fashion as observed in wild-type embryos, unless they were positioned at the tip of the invaginating primordium. Hence, less than 25% of the PGCs eventually arrive at the mesodermal gonadal primordia.

### 3.6. Genetic interaction between Tao-L and the Tre1 receptor

**Figure 6.**
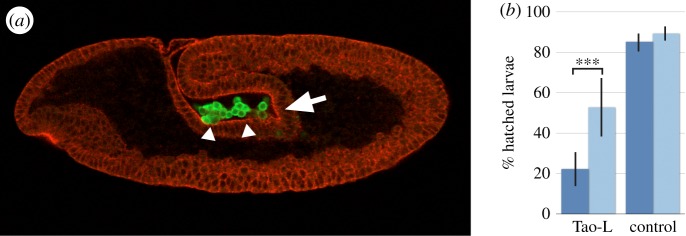
Reduction of the maternal *tre1* gene dose reduces the lethal effect of Tao-L overexpression. (*a*) *tre1* maternal mutant embryo (stage 9) showing that PGCs (arrowheads, visualized by anti-Vasa antibody staining; green) remain in the midgut/hindgut primordium instead of assembling in the tip region of the invaginating midgut primordium (arrow). Mouse PY20 anti-phosphotyrosine antibodies were used to outline all cells (red). Lateral view; anterior of the embryo is to the left, dorsal up. (*b*) Hatching of embryos (in %) that were derived from wild-type (dark blue) or heterozygous *tre1* mutant females (light blue) and received V3-driven ubiquitous Tao-L expression (‘Tao-L’). ‘Control’ refers to embryos from corresponding females that had a non-functional Tao-L expression transgene. Bars represent the mean value of between 12 and 21 independent egg depositions, each composed of 100 eggs. Standard deviation is indicated; asterisks (***) refer to *p* < 0.0001 (two-tailed *t*-test).

The PGC migration defect in response to enhanced Tao-S abundance at the posterior pole region of the embryos is reminiscent of the phenotype observed with *tre1* mutant embryos [5]. *tre1* encodes a GPCR that acts in a PGC autonomous manner [5,18]. In maternal *tre1* mutants, PGCs are trapped in the midgut or hindgut primordium (figure 6*a*) and, similar to the response to enhanced Tao-S expression in the posterior region, cells expressing the PGC marker protein Vasa remain at the surface of the embryo [5] (R.P. 2008, unpublished data). In addition, most *tre1* mutant PGCs remain trapped in the gut primordium [18], and the epithelial transmigration of the PGCs and their migration to the gonads is affected as observed in embryos after Tao-S expression in the posterior region of the embryo (R.P. 2008, unpublished observation). Based on these observed similarities, and as Tao-S acts as a putative Tao-L antagonist, we asked whether Tre1 functionally interacts with Tao-L, which is the one *tao-1* component present after gastrulation in PGCs [16].

In order to assess their possible functional interaction, we established a genetic interaction test system based on V3-GAL4-dependent maternal Tao-L expression in embryos derived from heterozygous *tre1* mutant females. Embryos obtained from heterozygous *tre1* mutant females develop into normal-looking embryos and larvae [5] (R.P. 2008, unpublished data), whereas V3-GAL4-dependent overexpression of Tao-L in otherwise wild-type embryos reduces the hatching rate of larvae to less than 25% (figure 6*b*). However, when the maternal gene dose of *tre1* was reduced in the offspring of heterozygous *tre1* females, the Tao-L-dependent lethal effect was significantly reduced (i.e. the hatching rate of larvae increased from 25% to more than 50%; figure 6*b*). This rescue of the Tao-L-induced effect suggests that Tao-L and the GPCR Tre1 are components of the same genetic pathway.

## 4. Discussion

We provide evidence that *tao-1*, which encodes the single *Drosophila* member of the *tao-1* subfamily of conserved Ste20 serine–threonine kinases, carries a second function that is independent of the catalytic kinase activity which resides in the N-terminal half of Tao-L.

Tao-L limits the growth of microtubule ‘plus’ ends when entering the actin-rich cortex of cells [12]. This microtubule-mediated function of Tao-L can be interrupted by drugs that interfere with microtubule assembly, confirming the earlier results. By contrast, Tao-S acts in an actin-mediated manner, a finding that is consistent with its enrichment in the actin-rich cortex of the cells. The cellular readout of Tao-S activity, however, is different from the one of Tao-L, as reflected in different cell shapes and behaviours of cells in response to each of the two proteins. Tao-L causes lamellipodia-like cell protrusions and negatively regulates microtubule stability [12]. By contrast, Tao-S causes filopodia-like structures similar to those observed after reduction of *tao-1* activity by RNAi knockdowns or in response to a Tao-L mutation lacking the functional kinase domain [12] (R.P. 2007, unpublished data). These findings, and the observation that cytochalasin D and latrunculin A (which interfere with F-actin stability and actin polymerization) inhibited the Tao-S-inducible filopodia-like structures, but did not interfere with the formation of Tao-L-dependent lamellipodia-like structures—and that drugs such as nocodazole and vinblastine (which destabilize microtubules) inhibited the Tao-L but not the Tao-S effects of cells—suggest that the Tao-1-dependent interplay between microtubule ‘plus’ ends and the actin-rich cell cortex [12] depends on two distinct functions. Both functions are exerted by a single gene, which encodes two proteins with different cellular functions, as reflected in the different cell shapes and cell behaviours caused by their expression in tissue culture cells.

We found that Tao-S antagonizes Tao-L activity and depends on F-actin, similar to what has been described for TESK1 [11], a LIM-related serine–threonine kinase [27] that participates in the reorganization of the actin cytoskeleton by phosphorylation of cofilin [27]. TESK1 inhibits Tao-1 activity in a kinase-independent manner by an as-yet-unknown mechanism [11]. To understand how Tao-S could possibly antagonize the activity of Tao-L and how this interaction compares mechanistically to the action of other regulating factors (such as TESK1) requires further biochemical analysis.

Maternal Tao-L and Tao-S transcripts are ubiquitously distributed in the egg, but they are rapidly restricted to PGCs when they form at the posterior end of the embryo. Ectopic Tao-L or Tao-S expression in the anterior region of the embryo induces pole cell formation, as observed after the injection of posterior pole plasm [28] or the expression of key components of the pole cell determinant assembly system [29]. Tao-1-induced pole cells are morphologically indistinguishable from normal PGCs, but fail to express germline cell markers. These observations indicate that the formation of pole cells and their determination as PGCs are separable processes. The fact that pole cell formation by *tao-1* is not dependent on the kinase domain further confirms that *tao-1* has more than one cellular function, and at least one of them does not involve kinase activity.

Overexpression of Tao-S, but not Tao-L, interferes strongly with PGC migration. PGCs are characterized by an actin-rich cortex, a rounded morphology [6] and, as they migrate through the midgut epithelium, by cytoplasmic protrusions [7]. This switch in cytoskeletal architecture correlates with the cell shape changes in response to Tao-L and Tao-S expression in tissue culture cells, respectively. Based on this correlation, we conclude that in response to Tao-S, which is normally not expressed in PGCs after the pole cells have formed [16], PGCs fail to properly undergo this cytoskeletal transition and, as observed in tissue culture, adhere to the surface of cells they are in contact with. The few PGCs that migrate are uncoordinated in both time and space. Furthermore, most of the migrating PGCs fail to arrive at their normal destination and, as observed with PGCs of wild-type embryos, they die if they fail to reach the gonad primordia. The death of PGCs, however, was surprising in view of an earlier study [16], showing that Tao-1 is necessary for the kinase domain-dependent activation of *sickle. sickle* causes cell death, a process which is normally repressed by maternal Nanos activity in the wild-type PGCs [16]. Hence, one would expect PGCs would survive when Tao-L activity is suppressed. The results imply, therefore, that posterior Tao-S overexpression does not interfere with Nanos-dependent suppression of the Tao-1 kinase activity, which in turn prevents *sickle* activation. The difference in phenotype caused in response to Tao-L and Tao-S expression, respectively, further confirms that the two proteins carry different functions.

Tao-1 and its known homologues in mammals, all of which contain the Ste20 kinase domain, have been shown to participate in a variety of cellular functions. In mammals, Tao proteins participate in the activation of the MAPK [30,31], phosphorylation of the Par-1 kinase (which regulates microtubule dynamics and cell polarity) [10], maintaining chromosomal stability by facilitating proper congression of chromosomes [15] as well as endocytosis of cadherin in dendritic spines [32]. In *Drosophila*, in addition to regulating microtubule plus-end growth in tissue culture cells [12] and apoptosis of germ cells [16], Tao-1 activity was shown to regulate adult brain development [17] and the growth of imaginal discs in which Tao activates the SWH pathway [13,14]. Furthermore, a Tao-1 mutation that lacks kinase activity was used to show that this activity is necessary for follicle cell morphogenesis by regulating the accumulation of polarity proteins at the plasma membrane and promoting Fasciclin 2 endocytosis [33]. Our findings suggest a function in cell migration as a new activity of Tao-1 and indicate that the *Drosophila* gene encodes two proteins which act in microtubule- and actin-mediated processes, respectively.

In addition to Tao-1, transepithelial migration of the PGCs through the posterior midgut epithelium requires attractive and repellant guiding activities provided by 3-hydroxy-3-methylglutaryl coenzyme A reductase [34,35], the lipid phosphate phosphatase 3 homologue Wunen [4,34–38], as well as the GPCR Tre1 [5]. The ligand that activates the receptor Tre1 in PGCs is unknown. Activated Tre1, however, feeds into an internal signalling process that involves the small GTPase Rho1, a member of the Rho family that plays a major role in reorganizing the actin cytoskeleton of cells (e.g. [39–42]). Our finding of a genetic interaction between *tao-1* and *tre1* is consistent with a model suggesting that the activation of Tre1 by one or several unknown external factor(s) participates in the control of PGC migration via a Tao-1-dependent rearrangement of the cytoskeletal architecture. In this model, reduction of the lethal Tao-L effect by reduced Tre1 activity leaves two options to explain the mechanism involved. One possibility is that activated Tre1 signalling causes activation of Tao-L. In this model, Tre1 signalling is required to set the level of the Tao-L kinase activity, which would be reduced by limiting Tre1 activity. This model implies that the two proteins interact directly or through other intermediate proteins. Alternatively, activated Tre1 acts by suppression of Tao-S. Derepression of Tao-S in response to reduced Tre1 activity would cause a reduction of Tao-L activity. Further molecular analyses will be required to dissect the Tre1 signalling cascade, to link its activity to components of the machinery that facilitates cytoskeletal rearrangements by Tao-1 and to elucidate Tre1 action on the two proteins encoded by the single *tao-1* gene.

## 5. Material and methods

### 5.1. Fly stocks

*Drosophila melanogaster* (Meigen) stocks were raised on standard cornmeal–yeast–agar medium at 22°C unless stated otherwise. EP(1)01455, Dp(1,Y)BSC136 and *ela*v^C155^-GAL4 were obtained from the Bloomington Stock Center, GE(1)01525, GE(1)02166 and GE(1)08166 from GenExel (Korea). V3-GAL4 was a kind gift from H. Bellen, *tre1*^Δ*EP5*^ from R. Lehmann.

### 5.2. Generation of *tao-1* mutants and expression constructs

*tao-1* mutants were generated as described, starting from EP(1)01455 [17]. Ectopic expression constructs were cloned from cDNAs LD40388 and LD45182 by PCR using ORF-specific primers and were fused in frame with the coding sequences for GFP, EGFP, VENUS, ECFP, mRFP-1 (Clontech, Saint-Germain-en-Laye, France) or mCherry [43]. Vectors contained GAL4-UAS and *actin5C* control elements. Transgenic flies were generated by pole plasm injection and P-element-mediated genomic integration [44]. RNAi fold-back constructs were directed against the region encoding the kinase domain and against the 5′ UTR of cDNA LD40388. Both constructs were cloned into pUAS-Ti.

### 5.3. Tao expression and mouse immunization

Antibodies were raised against a truncated protein of 357 amino acids, which contained the kinase domain of Tao-1. Antisera were produced in mice under standard conditions (three boosts in three months), with test bleedings made from the eye corner. Test sera from two mice were used at a dilution of 1/1000 for IHC and 1/20 000 for Western blotting.

### 5.4. Schneider S2 cell culture experiments

*Drosophila* S2 cells, cultured in Schneider's medium containing 10% fetal bovine serum and antibiotics, were transiently transfected using Effectene (Quiagen, Hilden, Germany) according to the manufacturer's protocol. Vinblastine, latrunculin A, cytochalasin D (Biomol, Hamburg, Germany) and nocodazole (AppliChem, Darmstadt, Germany) were added (1, 10, 5 and 10 µM, respectively) to the medium 1 h prior to fixation.

### 5.5. Antibody staining and *in situ* hybridizations

Antibody staining and RNA *in situ* hybridization in both embryos and ovaries were performed as previously described [45]. To visualize Tao-L transcripts antisense RNA probes were produced with SP6 from cDNA LD40388 cut with NsiI. Primary antibodies were mouse E7 anti-tubulin (DSHB, IA, USA, 1/50), mouse ADL67.10 anti-LaminO (DSHB, 1/100), mouse 2B10 anti-Cut (DSHB, 1/50), mouse 1D4 anti-Fasciclin II (DSHB, 1/50), Mouse PY20 anti-phospho tyrosine (Biomol, 1/400), rabbit anti-Vasa (R. Jauch, 1/5000), rabbit anti-Dlt (G. Vorbrüggen, 1/1000), rabbit anti-GFP (Synaptic Systems, Göttingen, Germany, 1/1000) and rabbit anti-RFP (Rockland, Gilbertsville, PA, USA, 1/1000). To mark the F-actin, phalloidin conjugated either to Alexa 568 or Alexa 647 was used (Invitrogen, Darmstadt, Germany; 2 units/sample). Anti-mouse and anti-rabbit antibodies coupled to Alexa 488, 568 or 647 were used as secondary antibodies (Invitrogen, 1/500).

Anti-DIG and anti-FITC coupled to AP (Roche, Mannheim, Germany, dilution 1/5000) were used for RNA *in situ* detection together with NBT/BCIP (Roche) and FastRed/Naphtol-AsBiphosphate (Sigma) as colour substrates. DRAQ5 (Biostatus, Shepshed, UK, 1/500) and SytoxGreen (Invitrogen, 0.1 µM) were used to stain DNA.

### 5.6. Microscope image acquisition

Images were acquired using Zeiss LSM410 and Leica TCS SP2 AOBS confocal scanning and Zeiss Axiophot transmission microscopes (Zeiss, Jena, Germany; Leica, Mannheim, Germany). Objectives were 0.5NA 20× air and 1.4NA 63× oil on the LSM 410, 0.7NA 20× oil and 1.25NA 40× oil on the SP2 and 0.6NA 20× air on the Axiophot microscope, respectively. Colorimetric stainings were embedded in Canada balsam (Sigma) or Murray's clear (benzyl benzoate (Sigma)/benzyl alcohol (Sigma) 2/1) and images captured at room temperature using a Kontron ProgRes 3012 (Jenoptic, Jena, Germany) camera and imported into Adobe Photoshop 4.0. Fluorescent stainings were embedded in Mowiol 40–88 (Sigma), ProLong Gold (Invitrogen) or Murray's clear. For live imaging, dechorionated embryos were covered by a drop of fluor halo carbon oil (Voltalev 10S, Atochem, Pierre-Benite, France) and imaged at room temperature (20–22°C). Cells were imaged in full culture medium using Lab-Tek chambers (Nalge, Naperville, USA) at room temperature. Images were processed and assembled using Adobe Photoshop v. 7, NIHImage v. 1.63 and MacromediaFreehand v. 10 software. Scale bars are pixel exact for the Leica SP2 derived images, but had to be approximated when taken with the Zeiss microscopes.

## Supplementary Material

Tao_2014_Resub_Suppl_Fig

## Supplementary Material

ESM_Figure_Legends.doc
