# Supplementary figures and images for: Drosophila gene tao-1 encodes proteins with and without a Ste20 kinase domain that affect cytoskeletal architecture and cell migration differently
Source: Open Biol. 2015 Jan 14;5(1):140161. doi: 10.1098/rsob.140161 (PMC4313371; doi:10.1098/rsob.140161)

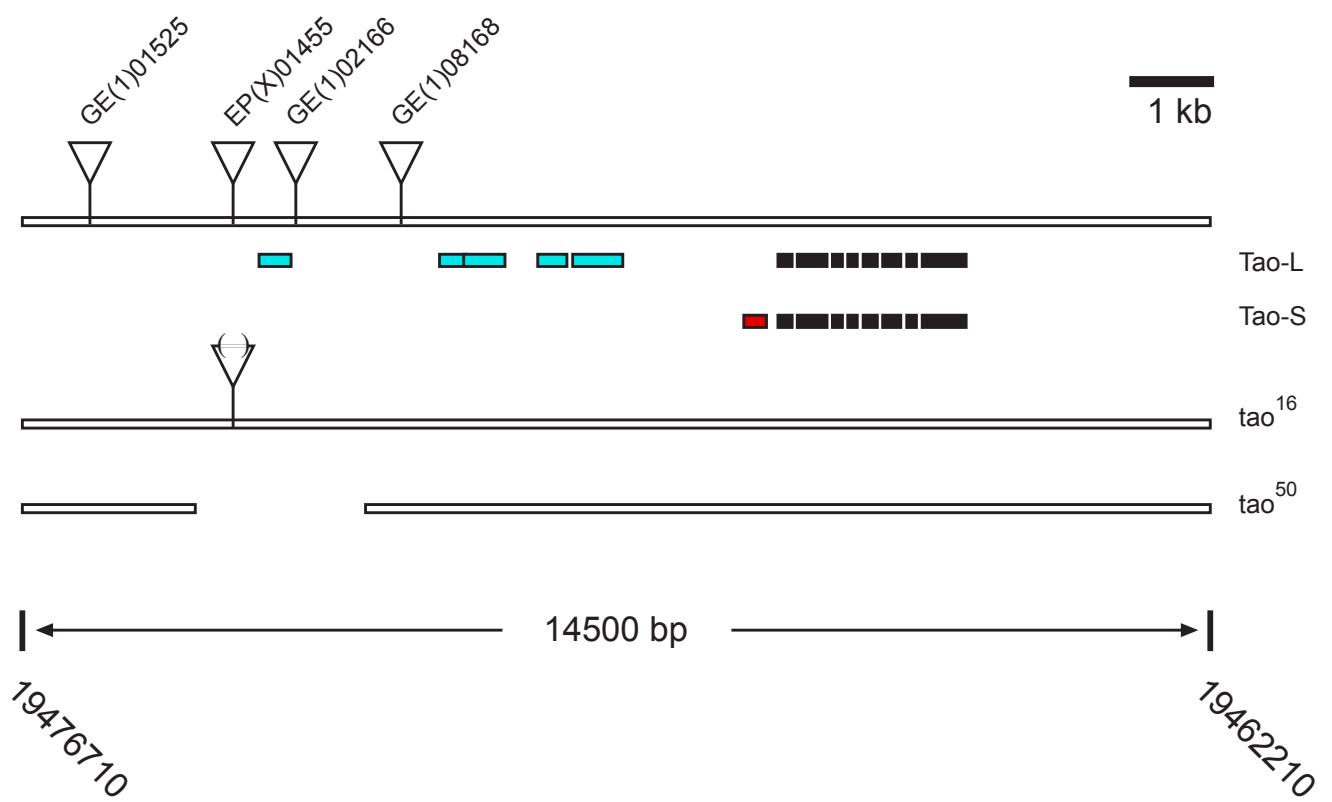

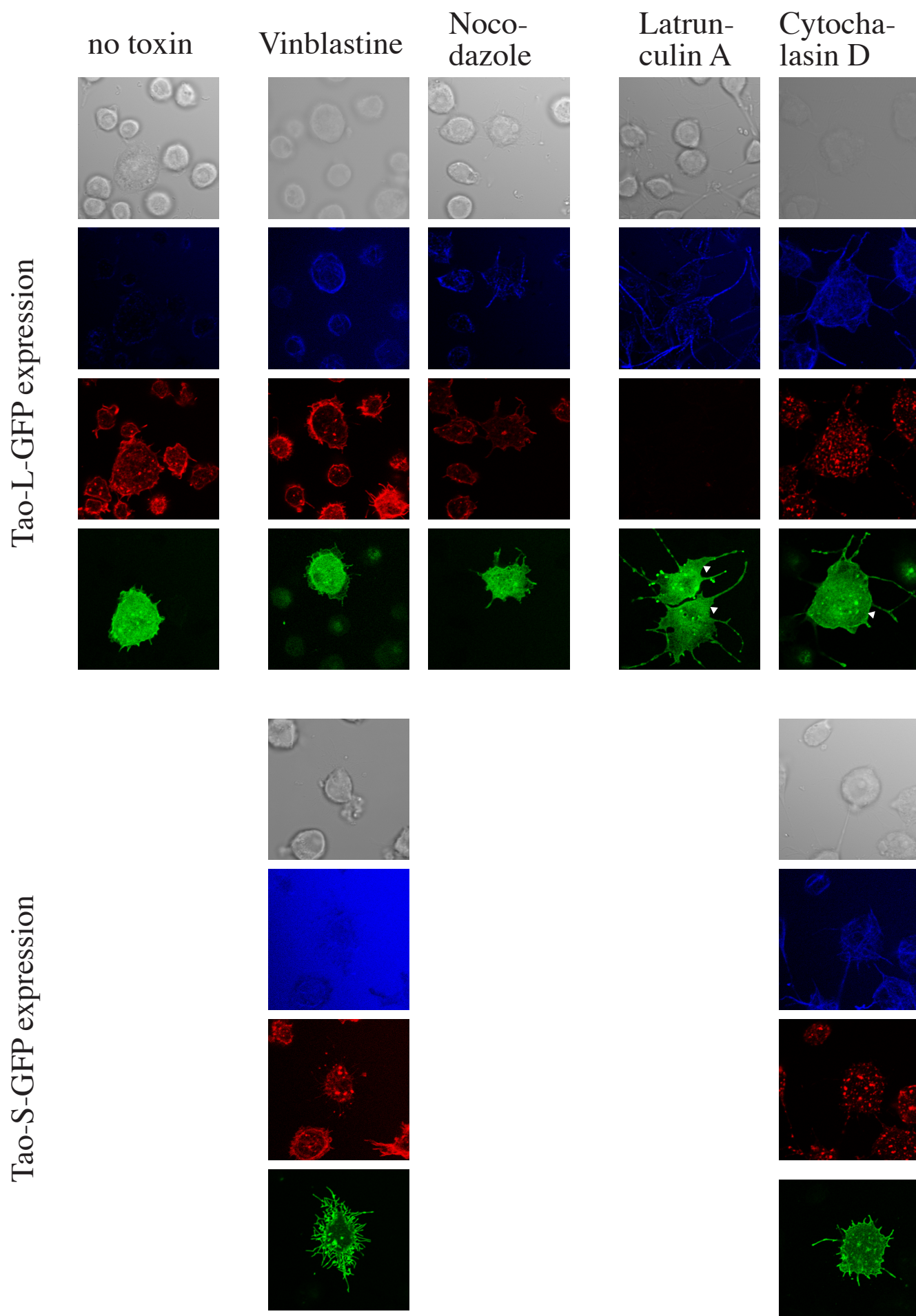

K56R-Tao-L-GFP

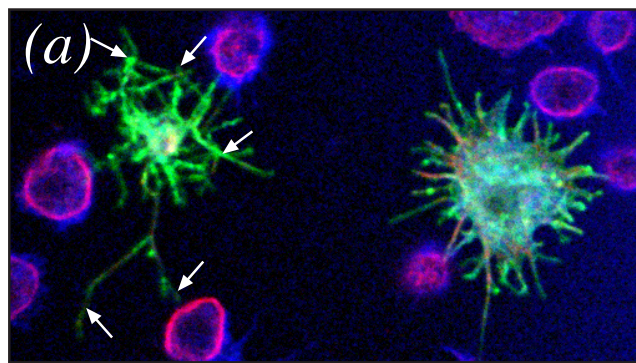

Tao-L-GFP

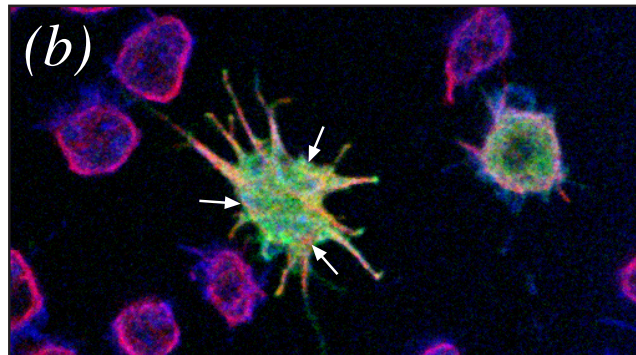

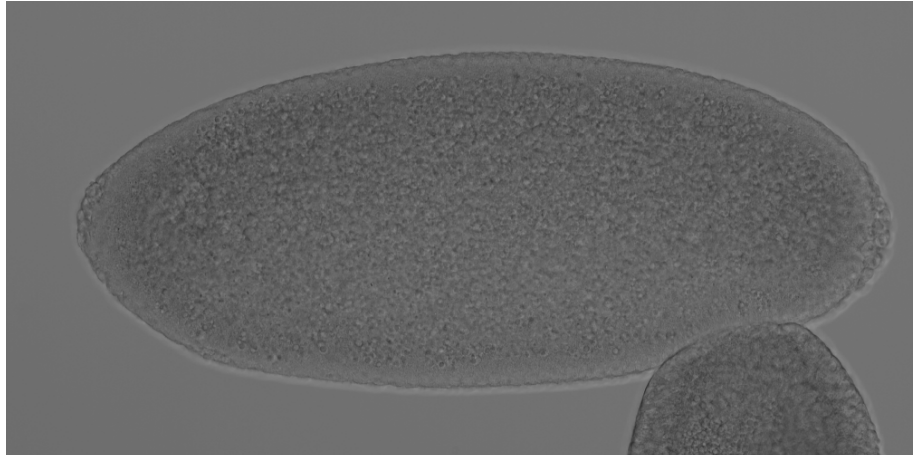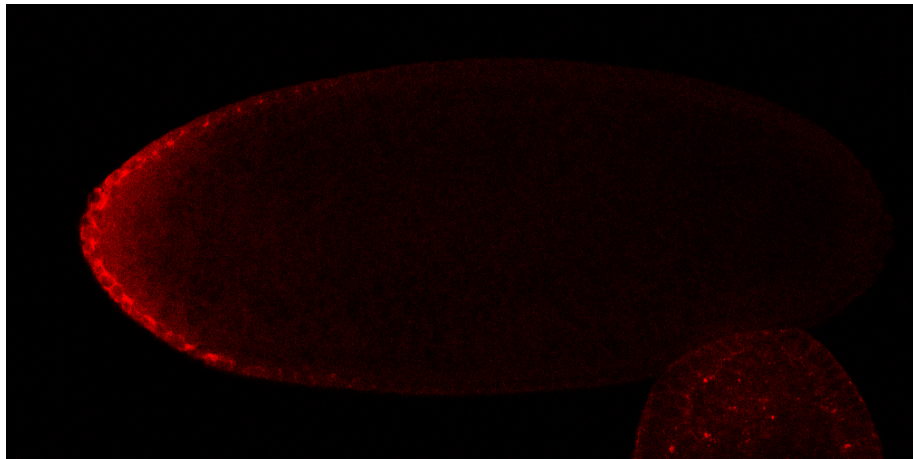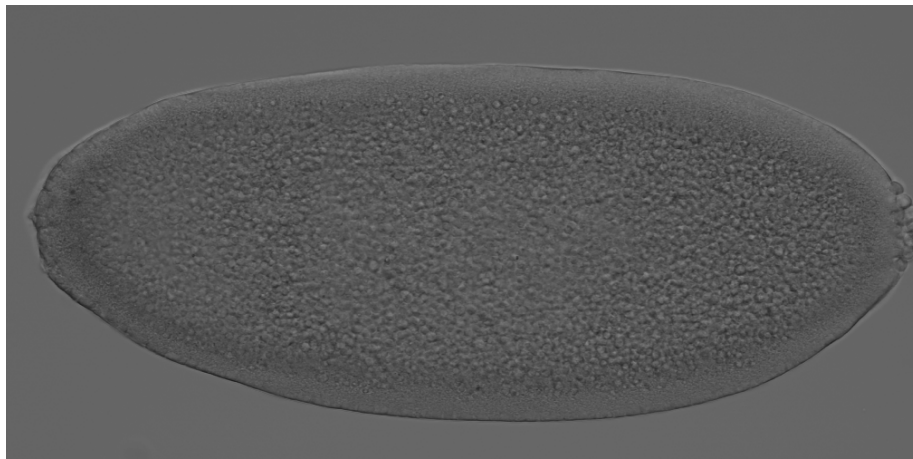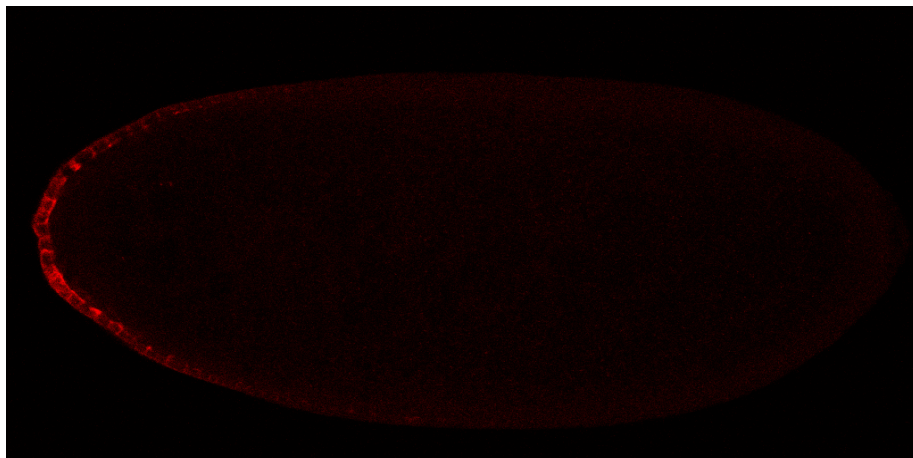

Supplement: Tao_2014_Resub_Suppl_Fig [file rsob140161supp1.pdf]
