# Supplementary material for: Drosophila gene tao-1 encodes proteins with and without a Ste20 kinase domain that affect cytoskeletal architecture and cell migration differently
Source: Open Biol. 2015 Jan 14;5(1):140161. doi: 10.1098/rsob.140161 (PMC4313371; doi:10.1098/rsob.140161)
Supplement: ESM_Figure_Legends.doc [file rsob140161supp2.doc]

**Electronic supplementary material**

**Figure Legends:**

**Figure S1:**

Genomic organisation of the *tao1* locus (position 19476710 -19462210 according to Flybase release 3.3). The positions of P elements EP(X)01455, GE01525, GE02166 and GE08168 are indicated by triangles. The deletions from jump out events from EP(X)01455 are an internal deletion within the P element for *tao^16^* and a small genomic deletion of 2077 bases for *tao^50^* and a deletion starting at the insertion point of EP(X)01455 uncovering the full tao1 locus. Exons coding for Tao-L and Tao-S are represented by bars; black refers to exons common to both proteins; exons specific for Tao-L are shown in blue, those specific for Tao-S in red.

**Figure S2:**

Tao-L-GFP expression (green, upper rows) in Schneider S2 cells stained with phalloidin-Alexa568 (red) and E7 anti-tubulin anti-mouse Alexa633 (blue). Transmission picture in grey and toxins as indicated above the pictures. Cytochalasin A and latrunculin A have a lower impact on the formation of Tao-L induced protrusions (white arrow heads) than the microtubule formation toxins nocodazole and vinblastine. Tao-S-GFP expression (green, lower rows) in Schneider S2 cells stained with phalloidin-Alexa568 (red) and E7 anti-tubulin anti-mouse Alexa633 (blue). Transmission picture in grey and toxins as indicated below the pictures. Vinblastine has a markedly lower impact on the formation of Tao-S induced small protrusions than cytochalasin A.

**Figure S3:**

Tao-L-GFP expression (green) in Schneider S2 cells stained with phalloidin-Alexa568 (red) and E7 anti-tubulin anti-mouse Alexa633 (blue). The K56R mutated form of Tao-L induces a cellular phenotype similar to the expression of the short form of Tao. *a*: Thin spiky protrusion are formed in cells expressing K56R-Tao-L-GFP. The left transformed cells body is detached from the cover slip and out of focus. Its thin protrusions (arrows) are in close contact with the glass surface, a typical effect of expression of Tao-S. *b*: expression of Tao-L-GFP induces more lamellipodia-like protrusions (arrows).

**Figure S4:**

Anterior expression of Tao-S-mCherry using maternal Gal4 (V3). Immuno localisation using anti-RFP antibodies and Alexa568 coupled secondary antibodies. Note that like with the corresponding expression of Tao-L (figure *5a,*b), anterior pole cells at the anterior end of the embryo are formed. Note that they do not develop into germ cells but undergo the pinching-off from the blastoderm cells. For details see text.

**Movie M1:**

Time lapse confocal microscopy of UAS_Tao-S-GFP driven by Actin-Gal4 in Schneider S2 cells. One individual confocal plane close to the cover slip was recorded. Time lapse factor is 900x i.e. 1s represents 15 min and the whole recording is 3 hours 10 min.

Thin protrusions are formed from those cells and attach to the substrate. Those protrusions remained immobile during the recording. Unattached protrusions are motile.

**Movie M2:**

Time lapse confocal microscopy of UAS_Tao-L-GFP driven by Actin-Gal4 in Schneider S2 cells. One individual confocal plane close to the cover slip was recorded. Time lapse factor is 900x i.e. 1s represents 15 min and the whole recording is 17 hours 30 min.

Tao-L expression results in flat and mobile lamellipodia which remain in contact with the surface of the dish and extend longer protrusions which are probing the cell surroundings. All cell attachments are highly dynamic and motile. The lower of the two cells expresses higher levels of Tao-L. Strong over expression of Tao-L causes protein aggregation over time.
